# Atherosclerosis as Mitochondriopathy: Repositioning the Disease to Help Finding New Therapies

**DOI:** 10.3389/fcvm.2021.660473

**Published:** 2021-05-04

**Authors:** Taisiia Shemiakova, Ekaterina Ivanova, Wei-Kai Wu, Tatiana V. Kirichenko, Antonina V. Starodubova, Alexander N. Orekhov

**Affiliations:** ^1^Institute of Translational Biomedicine, St. Petersburg State University, St. Petersburg, Russia; ^2^Institute for Atherosclerosis Research, Moscow, Russia; ^3^Department of Medical Research, National Taiwan University Hospital, Taipei, Taiwan; ^4^Institute of Experimental Cardiology, National Medical Research Center of Cardiology, Moscow, Russia; ^5^Laboratory of Cellular and Molecular Pathology of Cardiovascular System, Institute of Human Morphology, Moscow, Russia; ^6^Federal Research Center for Nutrition, Biotechnology and Food Safety, Moscow, Russia; ^7^Faculty of Therapy, Pirogov Russian National Research Medical University, Moscow, Russia; ^8^Laboratory of Angiopathology, Institute of General Pathology and Pathophysiology, Moscow, Russia

**Keywords:** atherosclerosis, mitochondriopathy, mitochondrial disease, chronic inflammation, mitochondrial therapies

## Abstract

Atherosclerosis is a complex pathology that involves both metabolic dysfunction and chronic inflammatory process. During the last decade, a considerable progress was achieved in describing the pathophysiological features of atherosclerosis and developing approaches that target the abnormal lipid metabolism and chronic inflammation. However, early events in the arterial wall that initiate the disease development still remain obscure. Finding effective therapeutic targets in these early processes would allow developing methods for disease prevention and, possibly, atherosclerotic plaque regression. Currently, these early events are being actively studied by several research groups. One of the processes that are being investigated is the development of mitochondrial dysfunction, which was demonstrated to be present in the affected areas of the arterial wall. Detection and characterization of mitochondrial dysfunction associated with several chronic human disorders was made possible by the improved methods of studying mitochondrial biology and detecting mitochondrial DNA (mtDNA) mutations. It was found to be involved in several key atherogenic processes, such as oxidative stress, chronic inflammation, and intracellular lipid accumulation. Mitochondrial dysfunction can occur in all types of cells involved in the pathogenesis of atherosclerosis: monocytes and macrophages, smooth muscle cells, lymphocytes, and the endothelial cells. However, therapies that would specifically target the mitochondria to correct mitochondrial dysfunction and neutralize the defective organelles are still remain to be developed and characterized. The aim of this review is to outline the prospects for mitochondrial therapy for atherosclerosis. We discuss mechanisms of mitochondria-mediated atherogenic processes, known mitochondria-targeting therapy strategies, and novel mitochondria-targeting drugs in the context of atherosclerosis.

## Introduction

Despite the tremendous efforts invested in the research of atherosclerosis and associated cardiovascular diseases and considerable progress in its diagnostics and management, the search for antiatherosclerosis therapies is far from finished. The available therapies are mostly focused on reducing known atherosclerosis risk factors, such as hyperlipidemia, alleviating the consequences of the disease and slowing down its progression (1). Moreover, available anti-atherosclerosis therapies do not target the earliest stages of atherosclerotic lesion development, where therapeutic intervention may be especially important for reducing the disease burden. During the recent years, the search for therapeutic targets mechanistically involved in the disease initiation received growing attention. One of the areas of interest is the chronic inflammatory process that may underly the disease development at the earliest stages (2). However, the atherosclerosis is known to be a multifactorial disease, which necessitates therapies that target a range of pathophysiological processes beyond inflammation. Furthermore, the list of genetic variants associated with atherosclerosis is constantly growing, making personalized medicine especially promising for prevention and treatment of the disease (3).

Among the events taking place at the early stages of the disease are inflammatory activation of both circulating immune and resident arterial wall cells, endothelial dysfunction, alteration of blood lipid profile, and oxidative stress. Strikingly, all these disturbances appear to be closely related to mitochondrial dysfunction, which may turn out to be the key link in the pathogenesis of atherosclerosis and a target for therapeutic agents (4). Mitochondria play a crucial role in, cellular metabolism, energy production and survival, especially in cell types characterized by high energy demands. In addition to energy production, mitochondria are involved in a number of vital cellular processes: calcium homeostasis, signaling function, which is partially performed through generation of reactive oxygen species (ROS), and initiation of apoptosis. Impairment of these mechanisms can lead to cellular dysfunction, uncontrolled ROS production followed by damage of cells and their surroundings, and initiation of the inflammatory response (5).

Accumulating evidence confirms the involvement of mitochondria in many pathological conditions, including cancers, cardiovascular pathologies, and neurodegenerative diseases. In this regard, mitochondriatargeting therapy has become a promising area of research (6). Human diseases caused by primary mitochondrial dysfunction, which is often associated with specific mutations and is hereditary, are termed mitochondriopathies. Besides that, general mitochondrial dysfunction can develop with age, and can also be associated with certain mitochondrial mutations and variants. Such dysfunction is observed in common human chronic diseases, including diabetes and some metabolic disorders. Accumulating evidence positions atherosclerosis as one of such diseases, with possible association of the pathology with certain mutations of the mitochondrial genes. Significant progress has been made in developing mitochondria-targeting approaches for anti-cancer therapies (7). It can be expected that mitochondria-targeting anti-atherosclerosis therapies will make comparable progress in the near future. A number of promising mitochondria-targeting agents is already available at pre-clinical and clinical stages of development. In this review, we will discuss the mechanisms of atherosclerosis development focusing on the role of mitochondrial dysfunction, and will outline the prospects of mitochondrial therapy.

## The Role of Mitochondria in the Pathogenesis of Atherosclerosis

Currently, it is believed that 2 processes play a decisive role in the pathogenesis of atherosclerosis: chronic inflammation and impaired lipoprotein metabolism. Atherosclerosis can be characterized as a chronic inflammatory condition, the main initiating factors of which are modified lipids that stimulate the innate and adaptive immune response (8). Atherosclerosis is associated with altered blood lipid profile, and disease severity correlates with the level of total cholesterol and low-density lipoprotein (LDL) cholesterol in the blood. However, the most dangerous are atherogenically modified forms of LDL that have undergone chemical changes, such as desialylation, oxidation, and glycation (9). It was shown that oxidized LDL could cause endothelial dysfunction by inducing the expression of adhesion molecules ICAM-1, VCAM-1 and P-selectins that promote growth and migration of smooth muscle cells, monocytes/macrophages, and fibroblasts. These alterations have a pro-inflammatory effect, since they affect the barrier function of the endothelium and render it adhesive for the circulating immune cells (10). A routine mode of entry of native LDL into the cells is mediated by a specific receptor, LDL receptor (LDLR). However, internalization of oxidized LDL is mediated by cellular scavenger receptors CD36, SR-A1, SR-A2, and LOX-1 that are responsible for the active uptake of oxidized LDL and, probably, other forms of modified LDL by macrophages (11, 12). Upon penetrating into the intima, monocytes differentiate into macrophages and start producing proinflammatory cytokines. Part of the macrophages actively participate in lipid uptake in the arterial wall, turning into foam cells that are abundantly present in growing atherosclerotic plaques. Transformation of macrophages into foam cells also decreases their ability to migrate, which leads to their accumulation in the atherosclerotic plaque and promotes local inflammatory reaction (13).

Oxidized LDL is involved in apoptosis induction pathways in macrophages and endothelial cells involving calcium-dependent mitochondrial pathways. These processes are mediated by cytochrome C and apoptosis-inducing factor (AIF) (14). It was hypothesized that mitochondria from cells deficient for LDLR could be involved in oxidative stress induced under conditions of a simulated state of hypercholesterolemia leading to the development of atherosclerosis. The study exploring this hypothesis demonstrated that mitochondria in *ldlr* knock-out mice produced more reactive oxygen species (ROS), were more sensitive to apoptosis, had lower antioxidant defense and were more susceptible to mitochondrial permeability transition (MRT) opening. The latter was associated with swelling of defective mitochondria and the outer membrane rupture, accompanied by the release of signaling molecules and apoptogenic factors (15).

It is currently well-recognized that mitochondria play a prominent role in the regulation of the inflammatory response: they control the expression of pro-inflammatory genes and the assembly of inflammasomes. Mitochondrial dysfunction also entails metabolic malfunction, which ultimately induces chronic inflammation (16). In addition, dysfunctional mitochondria are capable of increased ROS generation leading to the secretion of pro-inflammatory cytokines, such as IL-1β, by the affected cells (17).

Oxidative stress is one of the earliest and most significant signs of atherogenesis, which greatly contributes to inflammation and atherogenic lipid modification. Early atherosclerosis is characterized by a loss of balance between the oxidative and antioxidant systems of the cell. During the progression of the disease, the activity of antioxidant systems decreases mainly due to peroxynitrite-mediated inactivation of MnSOD2 and proteasome-mediated degradation of other enzymes (18). Increased production of mitochondrial ROS during lipid peroxidation leads to the formation of various reactive aldehydes. Among them are malondialdehyde (MDA), 4-hydroxy-2-nonenal (4-HNE), and γ-ketoaldehydes. Their formation is associated with various pathologies: neurodegenerative diseases, carcinogenesis, cardiovascular diseases, as well as aging-associated process. The danger of peroxidation products formation is related to their ability to form bonds with DNA and proteins, altering their structure and activity (19).

The loss of redox balance on the inner mitochondrial membrane can lead to a malfunction of the steroidogenesis process contributing to atherosclerosis development. Accumulation of ROS on the inner mitochondrial membrane can disrupt the mitochondrial cholesterol transporter, steroidogenic acute regulatory protein (StAR), which inhibits the delivery of cholesterol (necessary for steroid synthesis) from the outer to the inner mitochondrial membrane (20).

Normal mitochondrial function is critical for cell survival and is determined by the balance between the processes of mitophagy (autophagic degradation of mitochondria) and mitochondrial biogenesis (fission and fusion). The process of mitophagy helps eliminating defective mitochondria, which prevents their accumulation and associated malfunction of the cell and subsequent apoptosis (21). Mitophagy is therefore one of the barriers for ROS accumulation in damaged mitochondria. Atherosclerosis development is associated with deficient mitophagy, which contributes to progressive cell death, cell stress, and ROS accumulation, ultimately leading to the formation of a necrotic core and atherosclerotic plaque destabilization (22).

The increased generation of ROS during mitochondrial dysfunction causes damage to mitochondrial DNA (mtDNA), which is more prone to mutagenesis than nuclear DNA due to a difference in DNA packaging and repair systems. Accumulation of certain mtDNA mutations further contributes to mitochondrial dysfunction and atherosclerotic lesion progression, and is also involved in plaque destabilization processes. Mutations in mtDNA can lead to a decrease in the synthesis of respiratory complexes and weaken mitochondrial respiration in smooth muscle monocytes and macrophages (22). The effects of ROS have been studied in cell culture models that demonstrated damage to mtDNA, decrease in the amount of coding mtDNA, impaired mitochondrial protein synthesis, alteration of mitochondrial membrane potential and decrease in total ATP production in smooth muscle and endothelial cells (23). These events are likely to play a role in the development of atherosclerosis, which is known to be associated with changes in the mitochondrial genome, such as increase of mtDNA copy number, mtDNA methylation, and appearance of mutations (insertions, deletions, insertions) (24). However, the causative role of ROS in mtDNA damage was challenged by the results of some recent studies. Damaged mtDNA was detected both in circulating cells and in vascular cells before the appearance of other atherosclerotic signs (25). It is therefore possible that mtDNA damage is the primary event that induces excessive ROS generation, violation of the mitochondrial membrane potential and mitochondrial dysfunction followed by the release of cytochrome C and activation of apoptotic pathways. In addition, damaged mtDNA can be recognized by the body as an endogenous damage-associated molecular pattern (DAMP), which provokes the inflammatory response (26).

Mitochondria play a prominent role in cells with high energy consumption, such as many cell types of the cardiovascular system. In vascular endothelial cells, mitochondria act as important regulators of apoptosis and nitric oxide (NO) production. They can affect cell signaling and cellular response to stress, to which this cell type is rather sensitive (27). Endothelial cells, in addition to their barrier function, regulate the vascular tone, transport of blood plasma molecules, hemostasis, inflammation, and lipid metabolism. Intact endothelial barrier prevents penetration of circulating cells, such as monocytes/macrophages, into the vascular wall. Endothelial dysfunction is one of the earliest signs of atherosclerosis (28).

Dysfunctional mitochondria were observed in smooth muscle cells isolated from human atherosclerotic plaques. Such cells were characterized by the presence of mtDNA mutations, reduced mitochondrial mass and defects of ATP synthase (29). Impaired mitochondrial dynamics in smooth muscle cells of atherosclerotic plaques can lead to their proliferation. Fission and fusion of the mitochondria determines their morphology, and the effectiveness of ATP synthesis, oxygen consumption and the potential of the mitochondrial membrane (30). These processes are controlled by mitochondria-associated small GTPases, including the mitochondria 2 fusion protein (mitofusin 2, Mfn2), which reduces proliferation and promotes apoptosis of smooth muscle cells, which makes this protein a potential target for the treatment of atherosclerosis (31).

In macrophages, mitochondrial oxidative stress associated with atherosclerosis contributes to activation of inflammation through the NF-κB mediated pathway (32). This process is characterized by synthesis of pro-inflammatory cytokines, adhesion molecules, and growth factors, that, in turn, can trigger the inflammatory signaling (33).

Thus, mitochondria have a central position in the pathogenesis of atherosclerosis, mediating the most important aspects of the disease development. This fact allows positioning them as a promising potential therapeutic target.

## Mitochondrial Therapies

**Figure 1:**
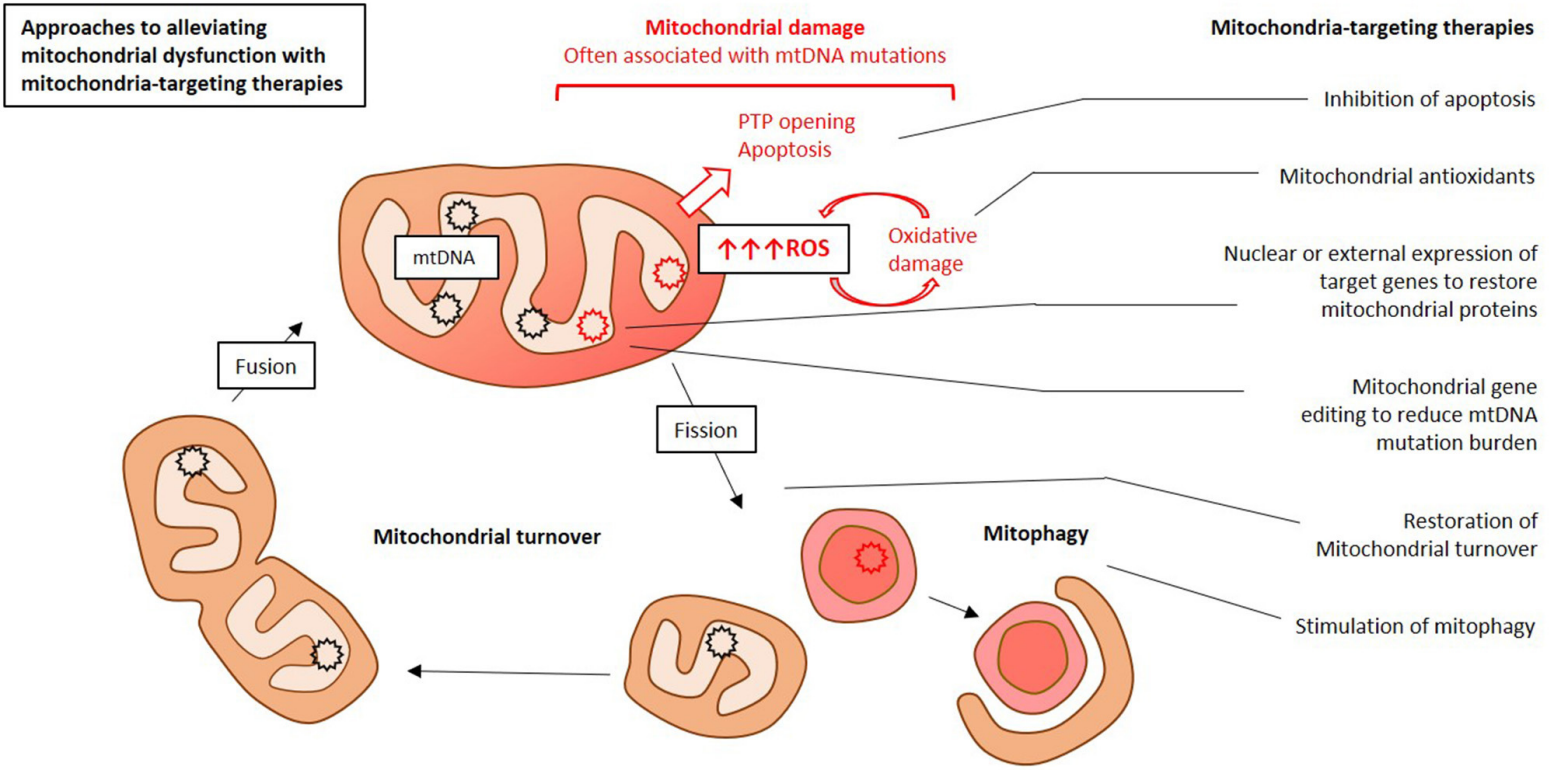
Simplified presentation of main approaches to mitochondria-targeting therapy for atherosclerosis. This Figure was drawn by E. Ivanova in Microsoft PowerPoint.

Existing mitochondrial therapies target both primary and secondary mitochondrial dysfunction. Primary mitochondrial dysfunction is caused by inherited defects of genes encoded by mtDNA or of nuclear genes encoding mitochondrial proteins (16). Primary mitochondrial dysfunction usually manifests in childhood, and is difficult to diagnose and treat. Curative treatment of such conditions would require gene therapy, hence replacement or suppression of a defective mitochondrial gene. These approaches still remain to be developed, and current therapy of primary mitochondrial dysfunction is mostly symptomatic (34). Secondary mitochondrial dysfunction can be acquired during the lifetime, and is commonly associated with chronic human diseases and age-related changes. Treatment of such conditions can involve both correction of mitochondrial functioning and stimulation of mitochondrial turnover to replace defective organelles with new and functional ones. Despite the different root causes of primary and secondary mitochondrial dysfunction, both are characterized by common general patterns of cell dysfunction and destruction. Among them are oxidative damage, disrupted calcium homeostasis and deficient ATP synthesis (35). Correction of these features of mitochondrial dysfunction can be achieved by therapies that act both inside and outside of the mitochondria (Figure 1) (34). Approaches such as diet, exercise, and vitamin supplementation [ubiquinone (CoQ10), vitamin C, E, biotin, zinc] are also known to be effective for alleviation of mitochondrial dysfunction (36).

There are several strategies for the development of mitochondria-targeting therapies. One of them is creation of molecules that selectively accumulate in the mitochondria. Mitochondrial antioxidants are able to alleviate the oxidative stress by selectively eliminating free radicals at the organelle level, where these agents accumulate. Another approach is designing molecules that aim at specific targets in the mitochondria. Gene therapy for correction of defective nuclear or mitochondrial genes is being currently developed. Cell therapy allows changing the number of copies of defective mtDNA and restoring the pool of functional genes. A new area of research is the use of proteasomes to eliminate and process defective mitochondrial components (37).

Studies of mtDNA variants associated with atherosclerosis have demonstrated that some of them correlate with the disease severity and onset timing. The ratio of normal to mutant mtDNA (the heteroplasmy level) appears to be a decisive feature and a potential marker of atherosclerosis-associated mitochondrial dysfunction. The threshold level of heteroplasmy (the level at which the mitochondrial dysfunction manifests itself) for several mtDNA variants can be considered as a marker of predisposition to atherosclerosis, and potentially be used for early diagnosis. Moreover, the severity of mitochondrial genome abnormalities can be reduced by increasing the ratio of wild-type mtDNA to mutant mtDNA. This possibility is currently being tested in preclinical studies (38).

### Gene Therapies to Restore mtDNA Defects

Mitochondria-targeting gene therapy is a novel and rapidly evolving research field. These methods can aim at blocking the mutated mtDNA genes and reducing their copy number or restoring the expression of mitochondrial genes of interest. Antigenomic therapy is based on the inhibition of mutated genes using anti-replicative oligonucleotides that reduce the accumulation of mutation-bearing mtDNA copies. The aim of such therapies is reducing the heteroplasmy level of the mutation of interest and restoring the respiratory chain activity (39). Deactivation of mutated mtDNA genes can also be selective, by designing vectors specific for certain mutations (40). Selective removal of mtDNA copies containing unique restriction sites has been reported in murine and human cell lines (37). Therapeutic application of gene engineering tools, such as transcription activator-like effector nucleases (TALENS) and zinc finger nuclease (ZFN)—site-specific restriction endonucleases has been explored (37). More recently, the clustered regularly interspaced short palindromic repeats (RISPR)/CRISPR-associated endonuclease (Cas) system, which revolutionized *in vitro* gene editing, was applied to altering mtDNA sequence (41). However, much work remains to be done to assess clinical feasibility of such approach.

Targeting delivery of gene editing machinery and vectors to the mitochondria remains a major challenge in these approaches. Mitochondrial delivery can be facilitated by using lipophilic cations, peptide nucleic acids (PNAs) of peptide nucleic acids. A promising approach is the use of lipid-based nanocarriers. Penetration through the outer and inner membranes of the mitochondria by these particles is ensured by the presence of hydrophobic and positively charged ligands that facilitate the process of endosomal transport. Lipid carriers are currently regarded as low toxic and highly selective transport systems (26).

An alternative approach to correcting the deficient mitochondrial genes is their nuclear expression, followed by cytosolic protein synthesis and vector transfection into the mitochondria. Despite some limitations of this method, it is already used to create animal models and is being introduced into clinical practice. Another tool for mitochondrial gene therapy is direct transfection. Its successful application leads to generation of mitochondria-targeting DNA carriers. The development of human mitochondrial gene vectors and cloning of the mitochondrial genome are currently ongoing. Positive results were reported for cloning of the murine mitochondrial genome in *Escherichia coli* (42).

In addition, injection of an adeno-associated virus carrying the required cDNA acts as a gene therapy that can promote restoration of mitochondrial dynamics by restoring the proteins responsible for this process, such as OPA1, a large mitochondrial GTPase, which is necessary for mitochondrial turnover. A successful example of using such therapy was reported for expression of OPA1 mitochondrial protein in a mouse model of dominant optic nerve atrophy (DOA), which accelerates the rate of degeneration of retinal ganglion cells. Replenishment of the amount of OPA1 protein was achieved by transduction of an adeno-associated virus carrying wild-type human OPA1 cDNA under the control of the cytomegalovirus promoter (43).

### Restoration of Mitochondrial Dynamics

A promising approach to alleviate mitochondrial dysfunction is removing defective organelles through the ubiquitin-proteasome system. It was shown that cardiovascular pathologies (including atherosclerosis), neurodegenerative processes, and malignant transformation are all associated with a decrease in the activity of ubiquitin proteasome system (UPS) and the accumulation of dysfunctional mitochondria. It is possible that proteasome inhibitors and proteasome rejuvenation pathways will prove efficient for restoration of this pathway through removal of harmful damaged organelles (44).

Another promising therapeutic strategy is activation of factors such as adenosine monophosphate kinase (AMPK) agonists, nitric oxide/cyclic guanosine monophosphate (NO/cGMP) stimulators, since mitochondrial biogenesis stimulation is known to be potentially cardioprotective (45). The cardioprotective effect of metformin, a widely used antidiabetic drug, demonstrated good results for treatment of postinfarction heart disease in a rat and dog models. Treatment with metformin showed protective effects mediated by AMPK signaling activation. The AMPK signaling cascade is responsible for the regulation of mitochondrial metabolism and stabilization of the cellular redox status. Metformin activation of AMPK improves cellular function during oxidative stress. An important contribution to this process is the inhibition of mitochondrial permeability transition pore (MPTP) opening and activation of the cellular antioxidant system (46).

In addition, activation of AMPK signaling pathways is capable of leveling mitochondrial fragmentation in the endothelial cells by inhibiting dynamin-related protein 1 (Drp1). Known pharmacological activators of these processes are metformin and resveratrol. Their protective action is mediated by inhibition of the inflammasome ER stress/NLRP3 due to a decrease in mitochondrial division (47).

In addition to metformin, there are other compounds that activate the AMPK pathway and can be considered as potential therapeutic agents for heart failure, among them 5-aminoimidazole-4-carboxamide-1-β-D-ribofuranosyl 5—monophosphate (AICAR), thiazolidinediones and statins (5-aminoimidazole-4 -carboxamide-1-β-D-ribofuranosyl, 5‘-monophosphate (AICAR), thiazolidinediones, and statins (45).

### Targeted Delivery of Drugs to the Mitochondria

Targeted delivery of therapeutic agents to the mitochondria is one of the priority directions of the current research. To date, several methods have been developed to deliver bioactive molecules to the mitochondria. First, lipophilic cations and mitochondria targeting peptides could be successfully used *in vivo*. A common lipophilic cation used for that purpose is triphenylphosphonium (TPP). This method allows delivering drugs that have naturally low affinity to the mitochondria because of their physical-chemical properties. Moreover, it reduces the off-target effects outside of the mitochondria therefore potentially reducing side effects. This strategy allows using small amounts of the active substance, which can be toxic, while its target concentration still remains at its maximum. At present, this approach is specific for organs with a high mitochondrial content (34).

Mitochondrial-penetrating peptides (MPPs) are lipophilic compounds that maintain a positive charge and have hydrophobic residues, which are the necessary features for successful penetration through the mitochondrial membrane. MPPs have already been shown to be effective in conjugation with a chemotherapeutic drug cisplatin (48). The peptides used for substance delivery, Szeto-Schiller (SS), act as antioxidants, protecting the mitochondria from oxidative stress. These peptides selectively bind to the inner mitochondrial membrane and accumulate in the matrix, and their penetration does not depend on the membrane potential (49).

Compounds based on guanidine, which can be small molecules or peptides, were shown to be capable of forming a bidentate hydrogen bond with cell surface phosphates, carboxylates and/or sulfates, allowing them to penetrate into cells and accumulate in the mitochondria. Due to the ability of guanidine-based compounds to stably bind to negatively charged proteins, these components can be successfully used for transporting proteins across the cell and mitochondrial membranes (50).

In addition, various nanocarriers, such as liposomes, micelles, and nanoparticles can be used for targeted substance delivery. The use of such delivery methods does not require chemical changes of the active substance, which is delivered in its native form. Nanocarriers are used to deliver low molecular weight substances: peptides, proteins, and small size nucleic acids. The advantage of nanocarrier therapy is the gradual release of the active substance, which reduces the side effects (51).

### Alternative Approaches

Non-pharmacological treatment of mitochondrial disorders includes gene therapy, ketogenic diet, and exercise. These methods are aimed to reducing cellular and mitochondrial oxidative stress, increasing the activity of the antioxidant systems, and stabilizing the mitochondrial membrane potential (27). Several studies have shown the effect of exercise on the mitochondrial genome and function. Endurance exercises contributed to a temporary (reversible) decrease in mtDNA deletions in human leukocytes (52). Mice lacking the mitochondrial polymerase gamma (POLG1) demonstrated activation of mtDNA repair pathways in response to exercise. Moreover, endurance exercise in mice restored mtDNA mutations and mitochondrial biogenesis involving p53 (53).

An interesting non-pharmacological therapeutic approach that has been tested for treatment of mitochondrial dysfunction is phototherapy (PT). The mechanism of PT is based on the selective action of photoreactive agents on the mitochondria and other subcellular compartments. This exposure leads to the absorption of light, which promotes the conversion of oxygen to ROS. Because of the key role of the mitochondria in the apoptosis initiation and ROS generation processes, such approach appears to be especially promising. Currently, the effectiveness of PT is being investigated for cancer treatment. PT was shown to induce hyperthermia and oxidative stress in cancer cells, which led to their death (54).

## Antioxidant Therapy for Atherosclerosis

The prominent role of mitochondrial oxidative stress in the development of atherosclerosis is currently well-recognized, and the potential protective capabilities of antioxidants are being actively studied. However, general antioxidant drugs failed to show any efficacy for atherosclerosis treatment. That may be due to the lack of redox status assessment before the treatment, as well as to the fact that the therapy effects were not selective for the mitochondria, which are the main source of the excessive ROS generation. Moreover, excessive antioxidant therapy may interfere with physiological functions of ROS that play an important signaling function in cells and tissues (55). Emerging mitochondrial antioxidant therapies may help overcoming this problem. These agents are targeted to the mitochondria, where they act selectively. The use of these agents for treatment of atherosclerosis is the subject of future research (56).

Human enzymes paraoxonase-2 (PON2) and PON3, located on the inner mitochondrial membrane, can also be an interesting candidate for antioxidant therapy of atherosclerosis. They are able to reduce mtROS-mediated apoptosis, which underlies their anti-atherogenic properties. The anti-atherosclerotic effect is based on peroxidation of cardiolipin and the release of cytochrome C, mediated by blocking the production of superoxide. Studies in a mouse model showed that PON2/3 was able to inhibit the production of superoxide due to its binding to ubisemiquinone (57). It is known that inhibition of PON2/3 activity causes the progression of atherosclerosis in humans and mice, and is associated with destabilization of atherosclerotic plaques. However, treatment with such agents may have side effects, such as tumor formation. Therefore, increased regulation of PON2/3 may have a cardioprotective effect, but further studies are required to assess its clinical utility.

Luteolin is a flavonoid-type antioxidant that is known to be protective against H_2_O_2_-induced oxidative stress by modulating the ROS-mediated P38 MAPK/NF-kB pathway. Luteolin alleviates mitochondrial dysfunction by suppressing the intracellular calcium increase and changing mitochondrial membrane potential. Moreover, it also regulates apoptosis by affecting p53 phosphorylation, decreasing the ratio of Bcl-2/Bax in the mitochondrial membrane, and cytochrome C release from the mitochondria, which leads to caspase 3 activation. The antioxidant properties of luteolin can help improving the endothelial function, thus making it a worthy candidate for atherosclerosis treatment (58).

Another potential tool for ROS neutralization for atherosclerosis treatment are peptide agents. Szeto-Schiller (SS) peptides are aromatic cationic peptides that have antioxidant properties and have been tested for alleviation of mitochondrial dysfunction. These agents have already been studied in the context of mitochondrial diseases. These small molecules can easily penetrate the cell and are not subjected to vesicular endocytosis. The main effect of SS peptides is the ability to prevent cell death and regulate lipid peroxidation. Peptide antioxidants appear to be promising therapeutic agents for treatment of diseases associated with oxidative stress due to their solubility, high efficiency, and low active concentrations compared to other drugs. They are easily synthesized, do not undergo peptidase degradation, have a relatively long half-life, and act on the inner mitochondrial membrane (59).

Another potentially useful antioxidant agent is melatonin, which was also shown to have cardioprotective properties. The mechanism of its antioxidant defense is mediated by the ability to stimulate mitophagy in macrophages, reducing the amount of ROS (60).

## Regulation of Mitochondrial Metabolism as Therapeutic Approach

Regulation of mitochondrial biogenesis can be considered as a therapeutic approach to reducing the endothelial dysfunction and vascular inflammation. Resveratrol was shown to mitigate vascular inflammation and induce mitochondrial biogenesis in a mouse model of type 2 diabetes and in aged mice. Further studies were performed in human coronary arterial endothelial cells (HCAECs). Due to the dependence of the amount of generated ROS on mitochondrial biogenesis, resveratrol also reduced mitochondrial oxidative stress (61).

One of the key risk factors for the development of atherosclerosis is the accumulation of modified lipids in macrophages of the vessel wall. Stimulation of cholesterol efflux from macrophages upon activation of the reverse cholesterol transport pathway can serve as a natural protective mechanis alleviating such accumulation (62). This process is closely related to the functionality of the mitochondria. This opens up opportunities for the treatment of atherosclerosis by stimulating mitochondrial metabolism in macrophages and enhancing the cholesterol outflow. MicroRNA-33 (miR-33) is known to be a post-transcriptional regulator of cellular and mitochondrial metabolism. It was reported that atherosclerotic plaques have elevated levels of miR-33. anti-miR33 therapy activates the genes responsible for enhancing mitochondrial respiration and ATP production, which contributes to cholesterol efflux. In *apoe* knock-out murine atherosclerosis model, such therapy led to a decrease of the lesions volume in the aortic sinus as compared with the control, although the amount of cholesterol in lipids remained unchanged (63).

It is known that PGC-1α is an important factor in the stimulation of mitochondrial biogenesis with cardioprotective properties mediated by the improvement of the endothelial function. A correlation of PGC-1α polymorphisms and the development of atherosclerosis and its complications was reported (64). Thus, activation of PGC-1α may be a beneficial strategy in the fight against atherosclerosis.

## Mitochondria-Targeting Anti-Inflammatory Therapy for Atherosclerosis

One of the key factors in atherosclerosis is the inflammatory processes, in which NLRP3 plays a prominent role, together with signal transduction pattern recognition receptors (PRRs). NLRP3 induces the inflammatory processes by releasing IL-1β and caspase-1, which are involved in atherogenesis, and are known plaque-destabilizing factors. Mitochondria are involved in the initiation of NLRP3 assembly as the main ROS generators and sources of damaged mtDNA. It was shown that blocking mtROS formation inhibited the assembly of NLRP3 inflammasomes (65). In addition, mitochondrial damage was directly associated with NLRP3. Mitochondrial cardiolipin was able to bind to NLRP3, and its lower level during mitochondrial membrane damage inhibited NLRP3 activation (66). Thus, targeting NLRP3 can potentially have anti-atherosclerotic effects that should, however, be evaluated by the future studies.

Salidroside is a phenylpropanoid glycoside that shows antiatherogenic activity in mice deficient for the LDL receptor. The effect of salidroside is mediated by an improvement of the endothelial function due to the activation of endothelial NO synthase (eNOS) by enhancing the phosphorylation of eNOS-Ser1177 and reducing the phosphorylation of eNOS-Thr495. Salidroside also exhibits anti-inflammatory effects due to the involvement of the AMPK/PI3K/Akt/eNOS pathway, leading to a decrease of cellular ATP, increase of the ratio of AMP-ATP balance in the cell, and reduction of the mitochondrial membrane potential (67).

The anti-atherogenic effect of anti-inflammatory cytokines, for instance, IL-35 and IL-10, is conveyed by their ability to block acute activation of the endothelial cells. IL-35 and IL-10 are able to suppress the production of mtROS, which contributes to activation of the innate and acute immune responses. These observations position IL-35 and IL-10 and agents enhancing their signaling as potential therapeutic agents (68).

## Nanotechnologies in Anti-Atherosclerosis Therapy Development

Nanotherapy and the use of nanoparticles (NP) is one of the emerging therapeutic approaches. In 2013, Marrache et al. (69) developed a mitochondria-targeting synthetic high-density lipoprotein that mimiced NP for treatment of atherosclerosis. This high-density lipoprotein (HDL) particle has a core of biodegradable PLGA and cholesteryloleate, which is coated with a phospholipid layer with TPP cations facilitating the delivery to the mitochondria, and the mimetic apolipoprotein A-I (apoA-I) peptide L-4F. It was shown that HDL-like nanoparticles could be delivered to the mitochondrial matrix and intermembrane space, with a small number of particles localizing on the inner and outer mitochondrial membranes. Distribution of the nanoparticles in the heart was studied in Sprague-Dawley rats 24 h after administration. The treatment resulted in a decrease of cholesterol and triglyceride levels (69).

The prospects for the therapeutic use of synthetic amorphous silica nanoparticles (SiNP) are currently widely studied. Initially, SiNP were developed for the delivery of genes and drugs for cancer therapy. However, it was found that the absorption of SiNP could lead to endothelial dysfunction, with disruption of endothelial cell homeostasis, *in vivo* and *in vitro* angiogenesis, apoptosis and endothelial cell necrosis, accompanied by oxidative stress. These observations indicate that SiNP can potentially be used for the development of anti-atherosclerosis treatments, although more studies are needed to better characterize their effects (70).

## Mitochondria-Targeting Gene Therapy for Atherosclerosis

Among the nuclear genes, it was possible to identify promising targets that could potentially be used to slow down the disease progression, including genes encoding for IL-1β, PCSK9, ApoE, LDR, and a number of lncRNAs and micro RNAs (miRNAs). Identification of target genes in the mitochondrial genome was less successful. Studies of mtDNA from atherosclerotic patients demonstrated that mtDNA damage and certain mtDNA mutations were associated with atherosclerosis. However, studying the mechanisms of these associations is challenging due to the difficulties in creating suitable animal models and lack of effective pathways for transfecting mammalian mitochondria. Mitochondrial genome editing techniques, such as mitochondrial zinc-finger nucleases (ZFNs), MitoTALEN, can potentially be used for mtDNA correction both throughout the body and in individual tissues or cell types (71).

Recent studies have demonstrated the role of miRNAs in the pathogenesis of atherosclerosis. MiRNAs are small regulatory non-coding RNA molecules. However, they are able to control the expression of more than 60% of protein-coding genes, and they themselves are controlled by processes such as DNA and histone methylation. These processes are known to be affected upon atherosclerosis development (72). Small interfering RNA (siRNA) are important regulators of mitochondrial function. The so-called mitomyRNAs are siRNAs of nuclear origin with mitochondrial localization. A number of miRNAs were found in the mitochondria from patients with various cardiovascular pathologies, including miRNA-696, miRNA-532, miRNA-690, miRNA-345-3p, miRNA-378a, miRNA-92a-2-5p, miRNA-143/145, miRNA-133a, miRNA-21, miRNA-1, miRNA-181c, miRNA-181a, miRNA-140, miRNA-15b, miRNA-16, miRNA-195, miRNA-424, and miRNA-21. Functionally, these siRNAs were found to be involved in regulating the energy metabolism, oxidative stress, glucose homeostasis, cell death processes, functioning of the electron chain complex IV, and induction of mitochondrial fission. Thus, siRNA mediating mitochondrial dysfunction can be used as biomarkers and therapeutic targets for atherosclerosis. MiRNA therapy has already been approved for conducting clinical trials (73).

Thanks to the improvement of genetic manipulation techniques, it became possible to consider the use of siRNAs as a therapeutic approach to atherosclerosis treatment. There are various ways to modulate the expression of siRNAs. The use of antisense nucleotides and/or siRNAs (ASOs or siRNAs) to regulate gene expression and improve cell function is currently under investigation. MiRNA inhibitors (also known as antagomiRs) that can inhibit miRNA function in mouse, primate and human cell models of diseases are currently being studied as possible pharmacological agents for regulating gene function. It is possible to inhibit siRNAs using specific blockers for target sites or using miRNA sponges that reduce the number of target sites available for binding (74).

## Atherosclerotic Plaque-Targeting Therapies

Direct therapies targeting the atherosclerotic plaques may be a promising and selective approach to the disease treatment. Especially dangerous are so-called unstable atherosclerotic plaques that are characterized by a thinned fibrous cap prone to rupture. This event leads to the release of highly thrombogenic material of the plaque necrotic core causing severe and life-threatening complications. Therefore, improving the plaque stability is an important task in managing atherosclerosis. The use of statins, antiplatelet, and antihypertensive drugs help reducing the risk of serious complications by alleviating the endothelial dysfunction and inflammation and reducing thrombogenicity. More specific approach is using immunomodulatory drugs, such as therapeutic antibodies against interferon-γ or recombinant IL-13. In addition, administration of peptides apolipoprotein B100, heat shock protein 60, or oxidized LDL may have a potential for stabilizing atherosclerotic plaques. However, the utility of these approaches for clinical practice could not be demonstrated with certainty (75).

One of the known factors of atherosclerotic plaque destabilization is apoptosis of vascular smooth muscle cells exposed to LDL. It was shown that active mitophagy helped avoiding this process. Stimulating mitophagy through known signaling mechanisms may therefore prove to be an interesting approach for atherosclerosis treatment relevant to plaque management. A study in human vascular smooth muscle cells demonstrated that mitophagy was activated in this cell type in response to oxLDL exposure. Silencing of key proteins regulating mitophagy, PINK1 and Parkin, impaired mitophagy flux, resulting in increased apoptosis of cells (76). A recent study highlighted the regulatory role of mitochondria-associated ER membranes (MAMs) and multifunctional MAM protein phosphofurin acidic cluster sorting protein 2 (PACS-2) in mitophagy. PACS-2 and Beclin-1 were shown to regulate the formation of contacts between the mitochondria and the ER leading to creation of mitophagosomes in vascular smooth muscle cells. Deficiency for PACS-2 resulted in impaired mitophagosome formation and mitophagy and increased apoptosis. These findings highlight MAMs as possible targets to improve plaque stability (77).

Medications that influence the regulation of the mitochondrial apoptotic pathway can be considered in the context of potential improvement of atherosclerotic plaque stability. Among such drugs is gypenoside, an extract of the medicinal plant *Gynostemma pentaphyllum*, which has a cardioprotective effect. Gypenoside can contribute to plaque stabilization through regulating the mitochondria of the endothelial cells and preventing their apoptosis. A recent study demonstrated that this compound regulated the expression of proteins involved in the PI3K/Akt/Bad apoptotic pathway, enhancing the expression of PI3K and p-Akt and suppressing the expression of p-Bad, cytochrome C, cleaved caspase 3, cleaved caspase 9, and PARP. In addition, gypenoside appeared to be an effective regulator of mitochondrial functionality, suppressing the expression of DRP1 and MFN2 proteins, which are involved in mitochondrial fusion and division, as shown on a mouse model. Possible beneficial effects of gypenoside for treatment of atherosclerosis are being currently studied (78). Atherosclerotic plaque-targeting therapeutic approaches are currently at the preclinical stage of development, and more studies are needed to identify potential drugs that can show efficacy at that level and evaluate their possible clinical use.

## Clinical Trials of Mitochondria-Targeting Drugs for Treatment of Atherosclerosis

Currently, treatment of atherosclerosis and associated disorders with mitochondria-targeting drugs is at the stage of preclinical research and, in some cases, clinical trials. The use of drugs based on generation of an endogenous antioxidant, ubiquinone, which is synthesized on the inner mitochondrial membrane, may turn out to be a promising therapeutic direction because of its effectiveness against lipid peroxidation and the ability to modulate redox processes. MitoQ is a synthetic analog of endogenous ubiquinone. It was shown that approximately half of MitoQ absorbed by cells is localized to the mitochondria (79). Treatment with MitoQ leads to the ubiquinol accumulation. The high hydrophobicity of MitoQ ensures the rapid penetration of the drug through the plasma membrane of the cell and its accumulation in the mitochondria, which is dependent on the mitochondrial membrane potential (80).

In 2018, the University of Nebraska has conducted a clinical trial (NCT03506633) with MitoQ to study its effect on the skeletal muscle mitochondria in patients with peripheral artery disease. The studied parameters included the level of oxidative stress. The double-blind study was conducted in patients aged 50 - 85 years, and the publication of results is expected in the near future (81).

A promising therapeutic strategy is the activation of factors such as adenosine monophosphate kinase (AMPK) agonists, nitric oxide/cyclic guanosine monophosphate (NO/cGMP) stimulators, since stimulation of mitochondrial biogenesis was shown to be cardioprotective (45).

Metformin is an FDA approved diabetes medication. In 2013, clinical trial (NCT01901224) of this drug in patients with peripheral artery disease was started, however, due to the cessation of financing, it was prematurely stopped (82).

Alteration of the blood lipid profile is a well-characterized feature and a risk factor of atherosclerosis. Hypercholesterolemia is associated with increased levels of ROS. In 2016, a study was conducted in patients with hypercholesterolemia to determine the therapeutic potential of simvastatin and ezetimibe in terms of improving the mitochondrial function. These drugs belong to the group of statins with antioxidant properties. Both drugs have shown their effectiveness: simvastatin as a lipophilic compound had a more pronounced effect, while ezetimibe a less pronounced. However, the combined use of both drugs showed the highest functionality. The positive effect of the drugs on the lipid profile, endothelial function, and mitochondrial function was explained by their inhibiting effects on ROS generation. Furthermore, the demonstrated effects included the reduction of plasma 8-Epi prostaglandin F2 alpha (8-epiPGF2a), oxidized LDL and lipoprotein-associated phospholipase A2, increase of the levels of SOD and CAT, and decrease of ICAM-1 in circulating monocytes (83).

Another clinical trial (NCT03980548) was aimed to study pharmacological inhibition of the mitochondrial fission protein, Drp1, which was shown to reduce the volume of atherosclerotic plaque and accumulation of macrophages in the plaque on *apoe* knock-out mouse model of wire carotid artery damage. Modulation of mitochondrial morphology and metabolism by means of Drp1 inhibition could be used to prevent atherosclerosis, reducing the activation and migration of monocytes. The study is currently at the recruiting stage (84).

An important role in the pathogenesis of cardiovascular diseases is played by the NO level. A decrease in its bioavailability, which can occur during oxidative stress, weakens endothelium-dependent dilation. It is possible to improve these functions using tetrahydrobiopterin (BH4), which is an important cofactor that regulates the functioning of eNOS. Studies in mice have shown that the pharmacological supplement BH4 increases NO bioavailability, alleviating inflammation and endothelial and vascular dysfunction. The level of BH4 can be controlled through increasing its production or decreasing its oxidative degradation (85). A recent clinical study (NCT03493412) was aimed to identify the effects of BH4 on vascular function, oxidative stress, and leg function in patients with peripheral arterial disease. The study is currently ongoing (86).

Valsartan-Entresto belongs to Ang II type 1 receptor blockers (ARBs). It was shown to have anti-atherosclerotic effects in patients with myocardial infarction, as well as in mouse models of atherosclerosis. The therapeutic effect of valsartan can be explained by its ability to modulate the activity of CD4+ T-lymphocytes, which has positive effects for stabilizing atherosclerotic plaques and regulating blood pressure (87). In 2015, the University of Minnesota began a clinical trial of Valsartan-Entresto (NCT02636283) in healthy volunteers and patients with peripheral artery disease in order to test its effect on the duration of walking without pain. In the course of the work, such indicators as mitochondrial and microvascular function, arterial elasticity, contributing to the pathogenesis of the disease, were measured (88).

NecroX (C25H31N3O3S · 2CH4O3S) is a mitochondrial antioxidant that has been shown to be effective against atherosclerosis *in vitro* and in animal models, reducing mitochondrial dysfunction. The drug passed 2 phases of clinical trials that have demonstrated its safety, and characterized its pharmacokinetics (89).

Astaxanthin is a membrane-penetrating, organic antioxidant of carotenoid nature. The antioxidant and mitochondria-protective effects of astaxanthin are demonstrated in cardiovascular disease models. Astaxanthin was shown to be safe both in healthy people and in patients, demonstrating anti-inflammatory effects and a decrease in lipid peroxidation. The drug passed the IV phase of clinical trials, improving lipid profiles of patients with coronary heart disease, treatment of patients with hyperlipidemia reduced the level of triacylglycerol (89).

In 2017, a clinical trial (NCT01755858) was conducted to study elamipretide, a mitochondria-oriented peptide that improves mitochondrial function when bound to the cardiolipin membrane. The study evaluated the effect of elamipretide along with percutaneous transluminal renal angioplasty on renal function in patients with atherosclerotic renal artery stenosis. It showed a reduction in the negative effects of ischemic damage after surgery (90).

## Conclusions

Accumulating evidence supports the hypothesis of the involvement of mitochondrial dysfunction in the pathogenesis of atherosclerosis. Impairment of the mitochondrial function leads to oxidative stress, inflammation, and metabolism changes, therefore contributing to different aspects of the pathology. Although mitochondria have been identified as attractive therapeutic targets in several human diseases, the development of mitochondria-targeting therapies is currently at the early stages. One of the challenges for developing mitochondrial drugs is targeted delivery to the organelles across the plasmatic and mitochondrial membranes. Several delivery methods have been developed, including lipid carriers and nanoparticles. These agents are currently being tested for therapeutic purposes. Antioxidant therapy for atherosclerosis has been extensively studied. It is likely that use of the antioxidants should be more specific and fine-tuned depending on the patient's redox status to improve efficacy and reduce off-target effect of interference with ROS signaling functions. Antioxidant therapy can be substantially improved by targeting the antioxidants to the affected mitochondria therefore alleviating the mitochondrial oxidative stress in a specific manner. Gene therapy targeting the mitochondria is a promising approach for treatment of atherosclerosis. A number of mtDNA mutations associated with atherosclerosis has been identified already, but more studies are needed to explore the possibility of using mitochondrial gene therapy for treatment of atherosclerosis.

## Author Contributions

AO, AVS, and W-KW: conceptualization. AO: resources. TS and EI: writing—original draft preparation and visualization. EI: writing—review and editing. AVS and AO: supervision. AVS and AO: project administration. AO: funding acquisition. All authors have read and agreed to the published version of the manuscript.

## Conflict of Interest

The authors declare that the research was conducted in the absence of any commercial or financial relationships that could be construed as a potential conflict of interest.
